# Effect of Brand Experience on Customer Engagement Through Quality Services of Online Sellers to Students in Bekasi

**DOI:** 10.3389/fpsyg.2021.801439

**Published:** 2022-01-14

**Authors:** Netty Merdiaty, Neil Aldrin

**Affiliations:** Faculty of Psychology, Bhayangkara Jakarta Raya University, Jakarta, Indonesia

**Keywords:** customer engagement, brand experience, service quality, student, Gen Z

## Abstract

Customer engagement refers to the emotional attachment a student experiences as a customer during repeated and ongoing interactions. Engagement occurs through satisfaction, loyalty, and excitement about the brand experience. Organizations engage customers at the point of behavioral change by exploring opportunities for emotional connection through continuous and consistent positive experiences. When customers engage with a brand experience, they feel emotionally connected and excited about the product and the service quality. This study’s purpose is examining the effect of brand experience on customer engagement by using service quality as a mediator variable; this research was conducted by collecting data from 254 students of the iGeneration born in 1995. Overall, 254 students participated in this study. Of them, 172 people or 68% of the total respondents in this study were women, and 82 people or 32% were males. The results show no direct effect of brand experience on customer engagement, and there is a role for service quality mediators that mediate brand experience and customer engagement. The results are discussed, and the implications for the organization are mentioned.

## Introduction

In the past few years, we have seen that research on customer attachment is on the rise. Initially, the attachment was in human resource management as a psychological connection to increase employee loyalty (Schaufeli et al., 2002); however, many researchers developed studies on attachment to the realm of marketing. Marketing has gone from transactional to relationships that emphasize the importance of interaction and value-laden, long-term customer relationships (Boulding et al., 2005; Rosenbaum et al., 2017; Sareen, 2018). In line with this shifting perspective, new concepts have emerged, including customer engagement (Vivek et al., 2014; Islam and Rahman, 2016). Customer engagement is a process not an end because it maps out the various customer behaviors and attitudes that result in positive, loyalty-focused brand consequences (Bowden, 2009; Verhoef et al., 2010; Hollebeek, 2011, 2015; Gummerus et al., 2012). According to Hollebeek et al. (2014) and So et al. (2014), measuring customer engagement can use two dimensions: cognitive and emotional.

The shift in these new concepts did not escape. The escalation of transactions over the internet, the development of information technology, and the influence of the Covid-19 pandemic contributed significantly to the popularity of online purchases that tend to lead to *iGeneration* students rather than traditional buyers. According to generation theory (Codrington and Grant-Marshall, 2004), there are five generations of humans based on the year of birth: *Baby Boomer*, born 1946–1964; Generation X, born 1965–1980; Generation Y, born 1981–1994, often called millennials; and Generation Z, born 1995–2010 and also called iGeneration, Net Generation, Internet Generation; and Generation Alpha, born 2011–2025. The focus on this study is on the iGeneration born in 1995; they are the generation that, since childhood, have known technology and are familiar with advanced gadgets that indirectly affect their personality. According to Kim and Ammeter (2008), young people are not only more familiar with e-commerce, but they also process website information five times faster.

Recently, competition between online sellers has become more intense, and online service quality sellers are receiving more attention than ever before. High-quality service has become a requirement among online sellers, and quality service helps companies get and keep customers engaged. According to Mittal et al. (1999), service quality is a focused evaluation of consumer perceptions of service quality components, such as interaction, physical environmental, and outcome quality. According to Sahin et al. (2011), brand experience is not a concept of emotional connection. Over time, a brand’s experience can produce an emotional bond, but emotions are just one internal result of stimulation that evokes the experience. Because brand experience differs from brand evaluation, attachment, and consumer pleasure, brand experience is also conceptually and empirically different from personality.

Marketing literature tends to see online brands as additional products or services that meet specific customer needs through interaction in a computer-mediated environment (Hoffman and Novak, 1996, 2009). A product can provide maximum emotional benefits to students as consumers; the brand must have a characteristic or uniqueness that distinguishes it from its competitors and provides a pleasant experience. Definition of brand experience is proposed by Brakus et al. (2009) as a bundle of feelings, sensations, cognitions, and behavioral responses elicited by brand-related stimuli that are brand identity elements. Marketing experts emphasize the emotive aspects of brand experience and subjective evaluation of brands, emphasizing the importance of brand personality (Okazaki, 2006), images [(Da Silva and Alwi, 2008a,b; Kwon and Lennon, 2009), or brand equity (Furrer et al., 2004; Christodoulides et al., 2006)].

This paper intends to examine the effect of the brand experience on customer engagement to quality services as mediators. The goal is important because customer attachment research is mainly fragmented and requires a general theory that is empirically verified, particularly among Generation Z.

## Hypothesis Development

### Brand Experience on Customer Engagement

According to Mollen and Wilson (2010); Hollebeek (2011), and Vivek et al. (2012), there is marketing research that defines customer engagement as the emotional, cognitive, and behavioral attachment of customers with brands. Customer engagement is embodied in four different sources of the value obtained from consumers: lifetime value (purchase), incentive referral, influence value, and knowledge value (Kumar and Pansari, 2016).

In line with the importance of customer attachment, the brand experience has also reached a significant place in recent marketing research, mainly due to its essential role in offering a competitive advantage to business organizations (Khan and Rahman, 2015). Attachment implies a two-party relationship (Vivek et al., 2014; Dessart et al., 2016) based on interactivity (Brodie et al., 2011; Hollebeek et al., 2019).

According to Carvalho et al. (2018), by buying one product, consumers take an active process to learn about the brand, which shapes the brand’s expectations. This process directs consumers to be more informed, connected, empowered, and active, and these experiences impact customer feelings positively. Customer attachment can be classified as a positive or negative feeling (Brady et al., 2006). Positive customer engagement includes positive consequences in the short and long term that are financial and non-financial for the company.

According to the research of Prentice et al. (2019), passengers’ experiences with airlines affects their emotional attachment and attitudes toward their choice, behavioral engagement with the airline, and ultimately loyalty behavior. The findings are consistent with those in the studies of Roberts and Alpert (2010); Kumar and Pansari (2016), and Pansari and Kumar (2018) although research exists in a variety of industry and study settings. Findings consistently show that customer experience with brands and related organizations is critical to engage customers to achieve customer engagement actively. The results of previous research are consistent with a view on the proposed hypothesis.

Hypothesis 1: It is suspect that there is a positive influence of brand experience on customer engagement.

### Brand Experience on Service Quality

There are divergent definitions of service quality in the existing literature. Some researchers study service quality as a general service evaluation. Service quality often reflects customer perception and value assessment of a product or service (Parasuraman, 1998), whereas others study it as a multidimensional construction shaped by service attributes. Service quality is a focused evaluation of consumer perceptions of service quality components, such as quality of interaction, of the physical environment, and of the results (Mittal, 1999).

Quality of service is widely recognized as an antecedent of customer satisfaction and behavioral intent that, in turn, leads to organizational profitability (Zeithaml et al., 1996; Alexandris et al., 2002; Wirtz et al., 2013; Shi et al., 2014; Kim et al., 2016). Its researchers argue that customers’ perceptions of service performance over each service experience determine the quality of a company’s services (Cronin et al., 2000).

Although the quality of service can be judged on a single meeting experience, another case in the brand experience is not limited to just one experience and one touchpoint only. However, it involves the experience from different touchpoints in different phases of the preconsumption journey, for example, when consumers consume the experience, alternative valuations, and anticipated experiences in brand organizations, including perceived sensations and memories of postconsumption experiences (Carù and Cova, 2003; Laming and Mason, 2014). The brand experience results from a series of interactions between brands and consumers during service meetings (Jiang et al., 2018). According to Sahin et al. (2011), customers need to have brand experience in marketing practices. This brand experience positively affects the quality of consumer–brand relationships.

The results of the research from Şahin et al. (2017) show that the relationship between brand experience and service quality is substantial.

The customer experience at each meeting is considered a quality snack that can emotionally improve customer feelings on the service manifested in purchasing behavior. Consistent with this view is the following hypothesis:

Hypothesis 2: It is suspect that there is a positive influence of brand experience on service quality

### Service Quality and Customer Engagement

It is imperative to engage customers and increase customer loyalty (Prentice and Wong, 2019). According to Chiou and Droge (2006), service quality generates overall satisfaction and trust and promotes purchasing intentions. As a result, in transaction relationships between brands and consumers, certain levels of trust and intention can increase a consumer’s willingness to continue relationships in the future or be attached. Verleye et al. (2014) assert that the overall quality of service significantly influences customer engagement behavior. On the other hand, Ahn and Back (2018) state that the customer brand experience is a positive and significant antecedent of customer engagement. On the other hand, the brand experience is related to the perceived quality of service. However, the research results from Prentice et al. (2019) show results in moderated mediation and *post hoc* testing or direct effects, suggesting that quality of service played a less significant role in customer engagement. The results provide empirical evidence of the gap between service quality and customer engagement, providing insight into the following service quality research. As such, we argue that, when a customer has better quality service, it means that he or she has a better brand experience and has the intention to engage with the product or brand.

The results of previous research consistent with a view on the proposed hypothesis are the following:

Hypothesis 3: It is suspect that there is a positive influence of service quality on customer engagement.

### Effect of Brand Experience on Customer Engagement With Service Quality as a Mediator

Brand experience includes cognitive and affective states (Bhat and Reddy, 1998; Mollen and Wilson, 2010), and several authors recognize the importance of both perspectives (Bridges and Florsheim, 2008; Hausman and Siekpe, 2009; Caruana and Ewing, 2010). Further evidenced by Gambetti and Graffigna (2010) and Brodie et al. (2011), brands are the most distinctive objects of engagement in business literature.

Perceived quality of service is defined as a global assessment or attitude relating to service superiority (Bitner et al., 2010). Recently Prentice and Loureiro (2018) conducted research closely from a customer’s perspective and examined how customers’ psychological desires, perceived benefits, and social values affect their engagement with brands and organizations, and they argue that customer-based antecedents better reflect their genuine engagement and willingness, leading to positive organizational outcomes in consumer behavior. Marketing shows that consumers no longer buy products and services, but rather buy experiences around what is sold (Morrison and Crane, 2007).

In their review, Prentice et al. (2019) state that the relationship of service quality is not so significant, but overall, research shows that customer-based factors are significantly related to customer engagement. In particular, brand experience has a significant direct and indirect effect on customer engagement.

Unlike most service research that models service quality as a predictor of customer engagement, this study proposes the quality of online seller services rated by students as customers acting as mediators in the chain effect of brand experience on customer engagement.

The results of previous research are consistent with a view on the proposed hypothesis:

Hypothesis 4: It is suspect that there is a role of mediator service quality on the effect of customer engagement with brand experience.

## Materials and Methods

### Participants and Procedures

A quantitative approach is used in research. The data analysis technique uses path analysis and the value of direct and indirect effects and regression analysis with intervening variables. To test the three hypotheses uses a quantitative approach, the data collection tool uses a psychological scale, and the research respondents are students in Bekasi. According to the research objectives, the analysis method uses the structural equation model (SEM) based on variance or variance based-SEM.

Respondents in this study were students in Bekasi with an average age of 23–30 years who were engaged in buying online, totaling 254 respondents, from a population of 750, a sample taken with table Krecie with research confidence 95% with alpha 5%. A total of 172 people or 68% of the total respondents in this study were women, and 82 people or 32% of the total respondents were male, so the total respondents were 254 people. All respondents were voluntary, and all respondents received approval of the form by providing information about the purpose of the study.

This project involves human subjects. The research protocol was approved and reviewed by academics. Ethical approval is not needed following applicable educational guidelines and regulations. Informed consent from the participants is implied through the completion of the survey.

### Measures

To collect research data, researchers used the Likert-type psychological scale. To measure customer engagement we used two dimensions: cognitive and emotional engagement, proposed by Hollebeek et al. (2014) and So et al. (2014), whereas dimensions of brand experiences suggested by Brakus et al. (2009) are sensory, affective, intellectual, and behavioral. For the service quality measure, according to Mittal et al. (1999), service quality is the focused evaluation regarding the consumer’s perception of quality components of service such as interaction, physical environment, and outcome quality. The researchers developed all scales and items rated on frequency ratings ranging from 1 (never) to 5 (always) or a five-point scale.

### Data Analysis Method

To describe the statistical methods and data using mean, median, and standard deviation to determine the normality of data, multivariate normality testing using SPSS 21 software and testing the hypothesis using data analysis methods were used; we modeled structural equations based on SEM (VB-SEM) variants using AMOS version 24 software. To analyze descriptive statistical data, we underlined the correlation between variables focused on describing or explaining variables. By looking at the correlation between research variables, it is expected to understand the three variables studied to test the hypothesis. Data analysis methods were used to model structural equations based on SEM-based variants (VB-SEM) using AMOS version 24 software.

This model is a set of statistical techniques that allows simultaneous examination of a series of relationships. In the SEM, variables that are not affected by other variables are called independent or exogenous variables, whereas other variables that are affected by other variables are called dependent variables or endogen.

## Results

**FIGURE 1:**
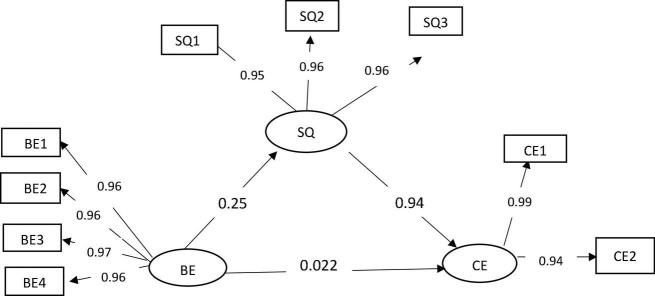
Path model. Customer engagement (cognitive and emotional), brand experience (sensory, affective, intellectual, behavioral), service quality (interaction quality, physical environment quality, outcome quality).

Based on the path analysis model in Figure 1, they use the AMOS version 24 program for processing data to form an estimation equation. After they are formed, a suitability test, goodness of fit, and hypothesis tests are performed.

Based on data processing results and acceptance criteria on model testing, match sizes determine the overall model’s predicted rate on correlation and a good covariance matrix. It can be seen with the chi-square value of 33,813, where the chi-square value result is small, the better, and the model is good (see Table 1).

**TABLE 1:** Model results.

Measurement conformity	Cut off value	Research result	Criteria
Chi-square (χ^2^)	≤48.561	33.813	Good fit
Probability	≥0.05	0.088	Good fit
Cmin/df	≥2.00	1.409	Good fit
GFI	≥0.90	0.973	Good fit
AGFI	≥0.90	0.949	Good fit
TLI	≥0.90	0.996	Good fit
NFI	≥0.90	0.991	Good fit
CFI	≥0.90	0.997	Good fit
RMSEA	≥0.08	0.040	Good fit

*Source: Hair et al. (2014).*

The value of chi-squares probability is 0.249 > 0.05, indicating that empirical data are identical to the theory/model. The value of the result, the root mean square error of approximation (RMSEA) is 0.040, indicating that the model is close to fit (see Table 1). Whereas the incremental suitability measure contrasting the proposing model with the base model looks very good by seeing at the value produced by the goodness of fit index (GFI) 0.973, where GFI is an index that describes the suitability of the overall model calculated from the predicted residual square of the model compared with the actual data. So the GFI > 0.90 (see Table 1) indicates that the model tested is suitable.

Adjusted GFI (AGFI) 0.949, Tucker Lewis index (TLI) is a cumulative conformity index by comparing the model tested with the baseline model. TLI is used to address problems arising due to the complexity of the model. The recommended acceptance value is TLI 0.996 > 0.90, and the normed fit index (NFI) measure compares the proposed and null models. The recommended value is NFI 0.99 > 0.90. The comparative fit index (CFI), 0.997, is also an incremental conformity index. The magnitude of an index ranges from zero to one; a value close to one indicates that a model has a good degree of conformity; this index is highly recommended to use because it is relatively less sensitive to sample size and less influenced by the complexity of the model. The recommended acceptance value is CFI 0.997 > 0.90

Influence analysis intends to see how strong the influence of a variable is with other variables indirectly, but directly variable BE low. The results of calculations of direct and indirect influence are shown in Table 2.

**TABLE 2:** Standardized total effect.

	BE	SQ	CE
SQ	0.249	0.000	0.000
CE	0.253	0.939	0.000

*BE, brand experience; SQ, service quality; CE, customer engagement.*

Through the calculation results in Table 3 and Figure 1, the direct effect of brand experience on service quality can conclude that brand experience has a direct effect of 0.272 (sig.). Similarly, the direct influence of service quality on customer engagement has an effect of 0.971 (sig.). Whereas between brand experience and customer engagement has a low direct effect, which has a value of 0.22 (0.452 > *p*). The following calculation results show that the indirect effect of brand experience on customer engagement through quality service is 0.934 (sig.). Because indirect influence is more significant than direct effects, it can be concluded that the service quality mediators play a full role in this study.

**TABLE 3:** Standardized direct and indirect effect.

	Direct effect		Indirect effect	
	β/γ	sig	γ	sig.
SQ ← BE	0.272	***		
CE ← SQ	0.971	***		
CE ← BE	0.022	0.452		
CE ← SQ ← BE			0.23	

*** P < 0,001.

## Discussion

Based on the results of the structural model analysis and the testing of the goodness of fit for this study, the effect of brand experience on customer engagement with service quality as a mediator gave the results that the research had a complete role—the statistical hypothesis testing of the effect of each variable to the other variables as follows. Of the four proposed hypotheses, only three were accepted (H2, H3, H4), and one was rejected (H1).

In hypothesis 1, there is no proven influence of brand experience on customer engagement. Although some studies say that brand experience has a significant effect on customer engagement, research conducted by Prentice et al. (2019) shows that passengers’ experience with airlines not only affects their emotional attachment and attitude toward the airline of their choice, but also their behavioral engagement with and on the airline. Finally, loyalty behavior, likewise, in research conducted by Roberts and Alpert (2010); Kumar and Pansari (2016), and (Pansari and Kumar, 2018). The research from Ahn and Back (2018) assert that customer brand experience is a positive and significant antecedent of customer engagement. This topic is particularly intriguing because the relationship between experience and engagement is controversial (Calder et al., 2009; Hollebeek et al., 2014). Given the intense focus on experience in modern marketing (e.g., Pine and Gilmore, 1998; Brakus et al., 2009), this can be considered surprising. However, other types of experiences may be better able to predict consumer behavior. According to Aldrin and Merdiaty (2019), today’s brand experience is no longer in demand, especially for young people or students (Gen Z).

In hypothesis 2, there is a proven positive influence of brand experience on service quality although research value is significant but not quite intense. This result is paradoxical to the finding research of Carrizo-Moreira et al. (2017); the study’s main conclusion is that brand experience is an essential antecedent of service quality, trust, satisfaction, and loyalty. Also, Devia et al. (2018) find that loyalty and brand experience significantly influence service quality to improve customer loyalty. Furthermore, loyalty and brand experience influence the improvement of service quality on customer loyalty.

In hypothesis 3, there is a proven positive influence of service quality on customer engagement. From the research we conducted, quality service relationships are very influential and significant to customer engagement firmly. This finding supports several prior studies that find service quality may lead to customer engagement: Ahn and Back (2018); Roy et al. (2018b); Lee et al. (2019). Roy et al. (2018a) investigated the impact of service convenience, fairness, and quality on customer engagement, finding that service quality has a significant effect on customer engagement. They are strongly supported by other research from Verleye and Aghezzaf (2013), asserting that overall service quality significantly influences customer engagement behavior. Also related to research from Abror et al. (2019), this study examined the link between service quality and customer engagement. The research finds that service quality is a significant and positive antecedent of customer engagement.

Finally, in hypothesis 4, there is a proven role of mediator service quality of customer engagement with brand experience, but it is very low. Unlike most service research that models service quality as a predictor of customer attitude and behavior outcomes, this study proposes that the quality of online seller services that students judge plays a role in mediation in customer attachment relationships. There is still little research on quality service as a mediator to customer engagement, so we compared the research from Prentice and Loureiro (2018) contending that customer-based factors are more reflective of customers’ volition to engage with a brand. Service quality is reflective of the cognitive assessment of the services provided by the brand organization. From the customers’ perspective, the firm should provide quality service to be competitive.

### Limitation of Study, Suggestion for Future Research

The study proposes that customer-based factors play a dominant role in engaging customers, and customer-based factors serve as mediators. There are several implications in this study. First, this study contributes to empirical customer engagement testing customer online seller-based factors among students with customer engagement. The results are extraordinarily challenging about the similar effects of customers and customer factors on customer engagement. Customer-based factors play a more prominent role in engaging customers. Second, the number of respondents is not too large, so it cannot be generalized to other students. A more significant number of respondents will give better and more accurate results. Therefore, future studies should test with a more significant number of respondents. Third, because respondents are students in generation Z, subjective possibilities must exist. It is hoped that future research can test more mature students or millennials or even customers of Generation X. Differences in views and cultures need to be considered, especially in the culture in Indonesia, and especially the Bekasi area needs to be the next concern in research.

It can also be caused by the ongoing Covid-19 pandemic, because of which changes occur in almost all sectors. Similarly, the local culture that influenced Gen Z decided to engage.

### Implication for Organization

Similarly, the findings of this study have important implications for the industry of online merchants in Indonesia and abroad. Regardless of the level of service offered to students, particularly among students, marketing efforts to engage customers among students need to be focused on improving customer experience based on customer psychology. In recent decades, service marketing researchers have widely promoted service quality in customer satisfaction and loyalty but have less involved the role of psychology in preparing its programs. The study shows that quality of service plays a role in mediation of customer-based outcomes. Although the results are identical or different with this study, they could be in a small portion or may contradict some of the results of previous studies; precisely, the effect of brand experience on customer engagement has positive but shallow results. It could be due to local cultural differences, considering that Indonesia has many cultures and cultural influences.

Furthermore, influence the way of thinking and making decisions for students to engage a customer. In terms of practical implications for the seller and providing an understanding of the attention to its customers, it is essential that the seller firmly understands the role of customer engagement for the continuity of its organization, for it requires awareness and attention for every employee in the seller’s organization to provide services following customer needs. Thus, seller organizations must pay attention to positive effects and experiences regarding the brand of an organization and the behavior of employees when interacting with customers. Because customer engagement does not show up instantly, seller organizations need to expand and improve other experiences that impact customer engagement. Hence, this research can guide seller organizations that want to increase their customer engagement.

## Data Availability Statement

The raw data supporting the conclusions of this article will be made available by the authors, without undue reservation.

## Ethics Statement

Ethical review and approval was not required for the study on human participants in accordance with the local legislation and institutional requirements. The patients/participants provided their written informed consent to participate in this study.

## Author Contributions

NM: study conception and design, data collection, and draft manuscript preparation. NA: analysis and interpretation of result. Both authors reviewed the results and approved the final version of the manuscript.

## Conflict of Interest

The authors declare that the research was conducted in the absence of any commercial or financial relationships that could be construed as a potential conflict of interest.

## Publisher’s Note

All claims expressed in this article are solely those of the authors and do not necessarily represent those of their affiliated organizations, or those of the publisher, the editors and the reviewers. Any product that may be evaluated in this article, or claim that may be made by its manufacturer, is not guaranteed or endorsed by the publisher.
